# 

**Published:** 1858-05

**Authors:** 


*Medical Society of South Western New York.*—This society will hold its annual meeting at Westfield, on Wednesday and Thursday, May 5th and 6th, at which meeting officers for the ensuing year will be elected. The Address by Dr. C. E. Washburn, on Wednesday evening. Dinner Thursday, at 12½ o'clock, for Physicians, their wives, and invited guests.

*Committee.*—Drs. J. Spencer, A. Cochrane, and T. D. Strong.

